# Molecular determination of abundance of infection with *Sarcocystis* species in slaughtered sheep of Urmia, Iran

**Published:** 2014

**Authors:** Farhad Farhang-Pajuh, Mohammad Yakhchali, Karim Mardani

**Affiliations:** 1 *Department of Pathobiology, Faculty of Veterinary Medicine, Urmia University, Urmia, Iran; *; 2 *Department of Food Hygiene and Quality Control, Faculty of Veterinary Medicine, Urmia University, Urmia, Iran.*

**Keywords:** Molecular analysis, * Sarcocystis*, Sheep, Urmia

## Abstract

*Sarcocystis* is one of the most prevalent parasites of domestic ruminants worldwide. This study was aimed to determine prevalence of *Sarcocystis *infection and molecular discrimination of *Sarcocystis gigantea *and *Sarcocystis medusiformis *infecting domestic sheep. Tissue samples from 638 sheep slaughtered at Urmia abattoir were randomly collected from February 2011 to January 2012. Genomic DNA extraction and polymerase chain reaction (PCR) was performed to amplify a 964 bp fragment of nuclear 18S rRNA gene. The PCR products were subjected to digestion with endonuclease *Mbo*II and/or *Mva*I for discriminating *S. medusiformis *and *S. gigantea*. Results indicated that the overall prevalence of *Sarcocystis* unspecified species was 36.83% (235/638) in which male (7.63%, 38/498) and female (35.00%, 49/140) sheep over 4 years-old had the highest prevalence. There was no significant difference between prevalence of macrosarcocysts and sex. Two macrosarcocysts forms were found as fat (27.90%, 178/638) and thin (8.93%, 57/638) in striated muscles. There was significant difference between frequency of macrosarcocysts and body distribution. Mixed infection with both fat and thin macrosarcocysts was also found in 11.13% (71/638) of infected sheep. There was no significant difference regarding the prevalence of mixed infection in both age classes. The PCR-RFLP patterns showed that fat sarcocysts were *S. gigantea* (29.31%, 187/638) and thin sarcocysts were *S. medusiformis *(7.52%, 48/638). It was concluded that ovine *Sarcocystis *infection was prevalent in Urmia and a combination of conventional methods and molecular study for sheep sarcocysts could be informative.

## Introduction

The genus *Sarcocystis* (Lankester, 1882) is an obligatory intracellular and widely distributed protozoan of the phylum apicomplexa in a broad range of vertebrates’ livestock and humans with about 130 species.^1^^-^^5^ The parasite is heteroxenous and has an obligatory two hosts, i.e. intermediate host with merogony and cyst formation in skeletal muscles (sarcocysts) of herbivores or omnivores and definitive host with sporogony and gamogony in carnivores or omnivores.^6^

Many animals are infected with one or more species of *Sarcocystis*. Sheep are intermediate hosts for *S. gigantea *(Railliet, 1886, syn. *S*. *ovifelis*), *S.* *tenella* (Railliet, 1886, syn. *S.* *ovicanis*), *S. arieticanis* (Heydorn, 1985), and *S. medusiformis *(Collins, Atkinson and Charleston, 1979).^5^ *S. gigantea *is distributed throughout the world while *S. medusiformisi* has been found only in Australia, New Zealand, and Iran.^6^^-^^8^ Two of these species,* S. gigantea* and *S. medusiformis *are transmitted by felids and are non-pathogens which develop macrocysts in striated muscles of sheep.^6^ Macroscopic cysts of these two *Sarcocystis* species are considered as causes of economic losses in the sheep industry.^9^ The heavily infected sheep meat by macrosarcocysts in Iran may be condemned as unfit for human consumption. Furthermore, nearly all investigations on ovine *Sarcocystis* infection are limited to the slaughter-house inspections without determination the prevalence of *Sarcocystis* species involved.^8^^,^^10^^,^^11^ The conventional method of distinguishing *Sarcocystis *species and combining these data with information on the life cycle are not suitable as a result of little morphological variation, high antigenic cross-reactivity, and time consuming.^12^ Therefore, the small subunit (SSU) rRNA gene has been extensively used to differentiate between apicomplexans and other eukaryotic species due to its abundance in the genome and its double feature of hypervariable regions interspearsed within highly conserved DNA sequences.^13^ Therefore, sensitive and specific molecular studies have been recommended to support detection and differentiation the parasites in the intermediate hosts that considered as a powerful tool for species-specific differentiation of ovine *Sarcocystis* species.^14^ In addition, there is no report concerning combine use of conventional methods and molecular techniques for comparison of *Sarcocystis* species in Iranian sheep. Therefore, the present study was carried out to determine prevalence of fat and thin macrocysts and molecular discrimination among *Sarcocystis *infecting sheep of northwestern Iran. 

## Materials and Methods

**Animals. **During the course of this study from February 2011 to January 2012, tissue samples (eso-phagus, diaphragm, and skeletal muscles) were randomly collected from 638 (498 male and 140 female) sheep slaughtered at the Urmia abattoir. All animals appeared healthy before being slaughtered. The age was estimated on the basis of eruption of permanent incisor teeth and the sex of each animal was recorded.^15^ The animals were categorized into two age classes, less than 4 years-old (n = 421) and over 4 years-old (n = 217), (Table 1).

**Fig. 1:**
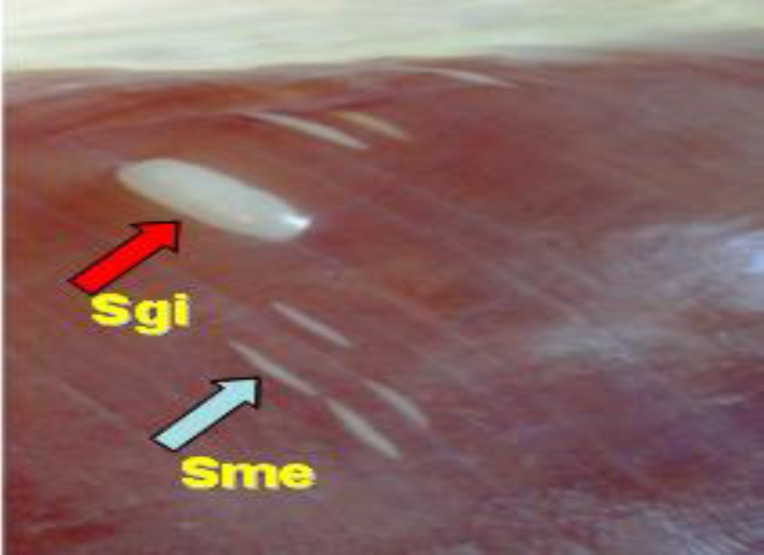
Fat and thin macrosarcocysts of *Sarcocystis gigantea* (Sgi, 5-10 mm) and *Sarcocystis medusiformisi *(Sme, 3.2 mm) in striated muscle of naturally infected sheep*.*

**Macroscopic and microscopic examinations. **The esophagus, diaphragm and skeletal muscles were thoroughly inspected for the presence of *Sarcocystis* macrocysts. Specimens containing macrocysts were separated, the cysts were excised from the tissue, and classified *in situ* based on their characteristic, namely size, shape and location, (Fig. 1). 

**Peptic digestion method. **Twenty gram of pooled muscles was incubated for 30 min at 40 ˚C in 50 mL of acid pepsin as digestion medium. The digestate was filtered through a fine meshed sieve into a tube, centrifuged at 2000 *g *for 5 min, and the sediment suspended in 0.5 mL of distilled water.^16^^,^^17^ The suspension was then microscopically examined for the presence of *Sarcocystis* bradyzoites under the light microscope at 400× magnification. In addition, further drops from the same solution were spread on glass slides, fixed, and stained with 1% Giemsa stain. The bradyzoites of *Sarcocystis* were measured using a micrometer eyepiece at 400× and 1000× magnifications. 

**DNA extraction. **For molecular analysis, soft cysts of the macrocysts were dissected, washed several times in 0.01 M phosphate buffered saline (pH 7.2), and stored at –20 ˚C until DNA extraction. Genomic DNA was isolated by modified phenol-chloroform method and stored at –20 ˚C.^18^

**PCR-reaction and RFLP analysis. **A pair of primers, Sar-sense: 5'- TTCTATGGCTAATACATGCG-3' and Sar-antisense: 5'- CCCTAATCCTTCGAAACAGGA -3' were used to amplify a 964 bp fragment of the 18S rRNA gene of *Sarcocystis*.^11^ The PCR-reaction was carried out in 25 µL reaction mixture containing 5 µL (100 ng) of genomic DNA (diluted 1:30), 0.3 µL of *Taq* DNA polymerase (Fermentas, Heidelberg, Germany), 0.6 µL of 10mM dNTPs (CinnaGen, Tehran, Iran), 0.7 µL of 50 mM MgCl_2_, 2.5 µL of PCR reaction buffer (10×), 1 µL of each primer (25 mM), and 13.9 µL of distilled water. The reaction was performed in a Bioer XP thermal cycler (Bioer Technology Co., Tokyo, Japan). The samples were subjected to an initial denaturation step at 94 ˚C for 5 min, followed by 35 cycles of 60 sec at 94 ˚C, 40 sec at 57 ˚C, and 60 sec at 72 ˚C, and a final extension step at 72 ˚C for 5 min. A total volume of 10 µL of each PCR product was analyzed by electrophoresis a long with positive and negative controls on 1.5% (w/v) agarose gel for approximately 90 min at 80 V and visualized by staining with ethidium bromide. 

To discriminate *S. gigantea *and* S. medusiformis*, RFLP method was employed. A total volume of 15 µL of digestion reaction containing 5 µL of the PCR product, 1 µL (1 U) of each of the restriction enzymes (*MboII *and/or MvaI, Fermentas, St. Leon-Rot, Germany), 1 µL of enzyme buffer (Fermentas, Heidelberg, Germany), and 8 µL double distilled water was prepared. The reaction tubes were incubated at 37 ˚C for 16 hr. The digested PCR products were run on 2% (w/v) agarose gel and visualized by ethidium bromide staining. Digested products for *S. gigantea* by *Mbo*II was expected to generate 722 and 242 bps in length, while *Mva*I was expected to produce two fragments of 901 and 63 bps in size. Digested products by *Mbo*II and *Mva*I was respectively expected to generate 901 bps in size and undigested product for *S.* *medusiformis*. 

**Statistical** **analysis. **Statistical evaluation was under-taken to compare obtained data with confidence interval of 95% using non-parametric *χ*^2^ test using Minitab (Version 16; Minitab, State College, USA). Probability values of *p *< 0.05 were regarded statistically significant.

## Results

**Macroscopic and microscopic findings. **The pre-valence of macroscopic and microscopic sarcocysts in slaughtered sheep has been shown in Table 1. The overall prevalence of *Sarcocystis* unspecified species, including macroscopic sarcocysts in slaughtered sheep was 36.83% (235/638). In rams and ewes, the prevalence of macrosarcocysts were 7.63% (38/498) and 35.00% (49/140), respectively (*p* > 0.05). Among the different examined organs, macro-sarcocysts were found to be the highest in the diaphragm (17.08%, 109/638) and the lowest in the skeletal muscles (1.72%, 11/638), (*p* < 0.05).

The macrosarcocysts occur as elongated cylindrical bodies and milky-white colored cysts embedded in the muscular tissues with length ranged from <5 mm to >10 mm (Fig. 1). Two inspected forms of macrosarcocysts were fat (27.90%, 178/638) and thin (8.93%, 57/638) macro-sarcocysts in striated muscles of which were large enough to discriminate by naked eye. Fat macro-sarcocysts were in the diaphragm with at least mean length of 5-10 mm (range: 2.50 to 15.00 mm, n = 100). The cysts were fully packed with banana shaped bradyzoites averaging 5.43 × 22.36 μm (range: 3.16 to 7.38 × 18.77 to 27.69 μm, n = 100), (Fig. 1). Thin macrosarcocysts occurred in esophagus and diaphragm with a mean length of 3.2 mm and width of 1.30 mm (range: 1.10 to 6.40 × 0.80 to 1.90 mm, n = 100). Also the bradyzoites of thin cysts were slightly smaller, averaging 4.21 × 17.29 μm (range: 2.95 to 6.38 × 16.41 to 23.15 μm, n = 100). Mixed infection with both fat and thin macrosarcocysts was also found in 11.16% (71/638) of infected sheep in both age classes (*p* > 0.05). Regardless of the age and sex, the rate of microsarcocysts infection was found to be 70.37% (449/638) by muscle squash (MS) method. In male and female examined sheep, the prevalence was 55.17% (352/638) and 15.20% (97/ 638), respectively, (Table 1). 

**Table 1:** Prevalence of macrosarcocyst and microsarcocyst of *Sarcocysts* species in slaughtered sheep at Urmia abattoir, Iran

**No. of examined sheep**	**Muscle inspection** ** (** ***n/N*** **, %)**							**PD ** **(%)**	**MS** **(%)**
	**Sex**		**Age (year)**		**Infected organs**				
	**Male**	**Female**	**< 4**	**> 4**	**Esophagus**	**Diaphragm** *	**Skeletal muscle**		
**638**	7.63	0	9.4	19.75	10.66	17.08	1.72	78.68	55.17
	0	35	0.16	7.52	1.72	4.55	1.41	17.71	15.2
**Total**	7.63	35	9.56	27.27	12.38	21.63	3.13	96.39	70.37

* indicates significant differences at *p* <0.05; PD: Peptic digestion method; MS: Muscle squash method; n: Animals infected with *Sarcocystis*; N: Total examined animals.

**Fig. 2:**
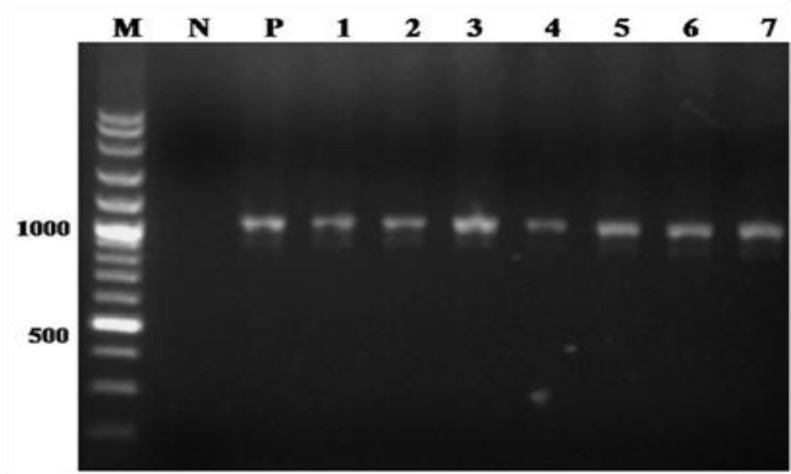
Agarose gel electrophoresis of 18S rRNA PCR products of macrosarcocyst of sheep (Lanes 1-7), Negative control (Lane N), Positive control (Lane P), 100bp DNA size marker (Lane M).

**Fig. 3:**
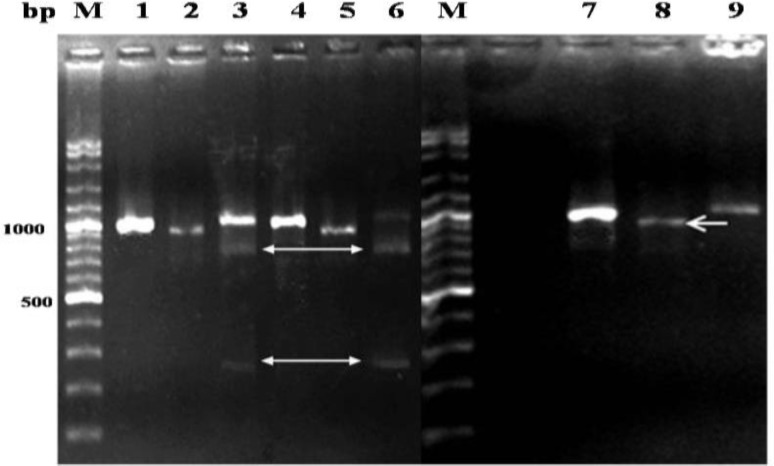
RFLP patterns of PCR products of *Sarcocystis* isolates from slaughtered sheep. Lanes 1 and 4: undigested 964 bp PCR products, *Sarcocystis gigantea*: lanes 2 and 5, *Mva*I digested PCR products*,* lanes 3 and 6 *Mbo*II digested PCR products; *S. medusiformis*: *Mbo*II Lane 8 digested PCR product;* Mva*I Lane 9 undigested PCR product), lane M: 100 bp DNA marker

**PCR-RFLP findings. **Of both identified *Sarcocystis* species (Fig. 2), 29.31% (187/638) fat sarcocysts were *S. gigantea* (Fig. 3). It was also shown that the thin sarcocysts which were less frequent (7.52%, 48/638) than the fat sarcocysts were *S. medusiformis*, (Fig. 3). The highest prevalence was 19.75% (126/638) in ewes (> 4 years-old) infected with *S. gigantea* and it was also 3.45% (22/638) in male sheep (> 4 years-old) which were infected by *S. medusiformis,* (Table 2).

**Table 2:** Prevalence and diversity of non-pathogenic *Sarcocystis *species (%) in both sexes and age classes of slaughtered sheep by using PCR-RFLP, (n = 638).

	***S. gigantea***				***S. medusiformisi***			
**Sex (%)**	Male		Female		Male		Female	
**Age (year)**	< 4	> 4	< 4	> 4	< 4	> 4	< 4	> 4
**Prevalence**	3.76	19.75	-	5.80	2.19	3.45	0.16	1.72
**Total**	23.51		5.80		5.64		1.88	

## Discussion

Sheep husbandry has been considered as the most important sector for food security supply of rural and sometimes urban areas in Urmia, Iran. Findings of this study revealed a moderate prevalence of macrosarcocysts infection in sheep. The prevalence of macrosarcocysts has been reported in slaughtered sheep of different parts of Iran including Fars province in south (57.70%), Shahrekord in southwest (18.63%), Khoram Abad in west (6.67%), Kerman in south (3.58%), in north of Khorasan province in northeast (0.04% ), and Ahvaz in southwestern Iran (0.0049%).^8^^,^^19^^-^^22^ The prevalence of *Sarcocystis* infection in sheep is variable throughout the world.^23^^-^^25^ The reason for high infection rate in intermediate hosts is the rearing of farm animals in close contact with carnivores which contaminate pastures with *Sarcocystis *sporocysts.^26^^, ^^27^

In the present study, the low prevalence of macro-sarcocysts in comparison with the prevalence of micro-sarcocysts in the examined animals may be due to the feline origin of macrosarcocysts as the contact between sheep and cats in the region were rare. Rommel found sporocysts in 15.00% of dog feces in Germany, while 5.00% of cats harbored sporocysts.^28^ Latif *et al*. noted that the abundance of macroscopic and microscopic sarcocysts was 4.10% and 97.00% in investigated Iraqi sheep, respectively.^26^ The high prevalence rate of microscopic sarcocysts in sheep indicates the importance of the infection for the intermediate hosts. The macrosarcocysts species are almost non-pathogenic but are responsible for economic losses because of the complete or partial rejection of the animal carcasses at slaughterhouses.^8^

The sex of examined sheep had significant effect on the prevalence. However in a study by Oryan *et al*., they reported that ewes had a higher prevalence of infection (61.07%) than males (38.93%).^8^ In addition, the prevalence of *Sarcocystis *infection in sheep increased as the age of animals increased. The age related distribution of *Sarcocystis *infection in age group <4 years-old was similar to that reported previously by Oryan *et al*.^8^ Also, molecular differentiation of ovine sarcocysts species showed the same pattern of age related prevalence of *Sarcocystis *infection for both identified *Sarcocystis *species.^8^ This difference might be due to the much higher mean age of male sheep at the time of slaughter and possibility of sarcocysts growing and high sensitivity of older sheep to gain infection.

The morphology of fully developed macrosarcocysts in the intermediate host varies among different *Sarcocystis *species and has been previously used to differentiate the species.^29^ In current study, two forms of macrosarcocysts were found in sheep muscles which were respectively discriminated as *S. gigantea* and *S. medusiformis* using molecular examination. Fat macrosarcocysts were commonly found in the diaphragm, while thin macro-sarcocysts were exclusively found in both esophagus and diaphragm of examined sheep. According to Oryan *et al*. and Heckeroth and Tenter, *S. gigantea* is predominantly found in oesophagus, larynx, and lingua muscles.^8^^,^^12^ They were also reported that *S. medusiformis* is commonly found in diaphragm, abdomen, and skeletal muscles. 

The results of a number of epidemiological studies on *Sarcocystis *infections in Iranian sheep by peptic digestion method (PD) procedure were in agreement with the results in the present study.^28^^,^^31^^,^^32^ The PD procedure gave the highest rate of infection and it was found more sensitive, simple, and rapid than tissue sectioning for detecting sarcocyst infections.^26^^,^^33^^,^^34^

 In current study, molecular investigation also evidenced both identified fat and thin sarcocysts respectively belonged to *S. gigantea* and *S. medusiformis*. It was also shown that the *S. medusiformis* were less frequent than *S. gigantea* in infected carcasses. Molecular findings indicated that the highest prevalence was in ewes infected with *S. gigantea* and male sheep infected with *S. medusiformis* over 4 years-old. In recent years, the advent of new molecular biological techniques has provided new diagnostic means for parasitic infections. The ssu rRNA gene amplification is specific way for discriminating *Sarcocystis* species.^35^ In earlier studies, PCR-RFLP method introduced as a tool with high specifity and susceptibility of discriminating *Sarcocystis* species worldwide. ^12^^,^^36^^-^^38^ PCR-RFLP was applied for the first to identify *S. gigantea *in Iranian sheep and recommended as easy and rapid method than DNA sequencing of discriminating between these species by Dalimi *et al*.^39^

In conclusion, the present work has demonstrated that *Sarcocystis *infection was common in sheep in Urmia. In addition, sheep husbandry is a sector of food supply for the rural and sometimes urban people and their health status is therefore important. Therefore, further investigations may reveal more information about economic effects of each type of this parasite in the region. On farms, it is important to reduce the sporocysts shedding in dogs and/or cats, which in turn will aid reduction of transmission within flocks. Finally a combination of conventional diagnostic methods and molecular identification of sarcocysts in sheep will be informative. 
